# Stereotactic radiosurgery for cerebral arteriovenous malformations: Evaluation of long-term outcomes in a single institute cohort

**DOI:** 10.1007/s00066-025-02461-5

**Published:** 2025-09-26

**Authors:** Luis Mohr, Philipp Lishewski, Markus Schymalla, Kerem Tuna Tas, Edgar Smalec, Rita Engenhart-Cabillic, André Kemmling, Maximilian Schulze, Khaled Elsayad, Fabian Eberle, Christopher Nimsky, Hilke Vorwerk, Klemens Zink, Ahmed Gawish, Sebastian Adeberg

**Affiliations:** 1 Department of Radiotherapy and Radiation Oncology, Philipps-Universität Marburg, Marburg, Germany; 2 Department of Radiotherapy and Radiation Oncology, University Hospital Marburg, Marburg, Germany; 3 Marburg Ion-Beam Therapy Center (MIT), Department of Radiotherapy and Radiation Oncology, Marburg University Hospital, Marburg, Germany; 4 University Cancer Center (UCT) Frankfurt—Marburg, Marburg, Germany; 5 Department for Neuroradiology, Marburg University Hospital, Marburg, Germany; 6 Department for Neurosurgery, Marburg University Hospital, Marburg, Germany; 7 LOEWE Research Cluster for Advanced Medical Physics in Imaging and Therapy, (ADMIT), TH Mittelhessen University of Applied Sciences, Giessen, Germany

**Keywords:** Vascular anomalies, Brain, Congenital abnormalities, Blood vessels, Obliteration

## Abstract

**Objective:**

The study we describe focuses on evaluating the effectiveness of linear accelerator (LINAC) stereotactic radiosurgery (SRS) in the treatment of cerebral arteriovenous malformations (AVMs). This treatment option is gaining interest due to the uncertainties associated with combined radiosurgical and endovascular treatments and the significant technological advancements in SRS. The primary goals of the study are to assess rates of obliteration (successful closure of the AVM) and rebleeding (the recurrence of bleeding posttreatment), as well as to identify factors influencing obliteration rates and to document any adverse effects associated with the procedure.

**Materials and methods:**

The study retrospectively analyzed data from 134 patients treated with LINAC-based SRS for cerebral AVMs. The patients were categorized based on their prior treatments: 50 had undergone partial embolization, 8 had received a combination of embolization and surgery and 1 patient had a surgical intervention. Furthermore, 75 patients had received no prior treatment. Kaplan–Meier survival analysis and log-rank tests were employed to calculate actuarial obliteration rates and annual cumulative bleeding rates following SRS treatment.

**Results:**

The study found that obliteration rates after SRS treatment increased over time, with 5‑year obliteration rates of 85.2% for grades I–II, 76.4% for grade III, and 62.1% for grades IV–V. Annual cumulative bleeding rates post-SRS were 1.5% for the first year and 0.7% for the second year. Interestingly, prior embolization did not affect the obliteration rate. The median time to obliteration was 36 months (range 7–162 months). Obliteration rates were significantly better in Spetzler–Martin (SM) grade I–II (85% at 5 years) compared to grade III–V (68% at 5 years, *p* = 0.01). Age, sex, and pediatric status had no statistically significant influence on AVM response to SRS. No radiation necrosis was observed in our cohort.

**Conclusion:**

This study contributes to the body of evidence supporting the effectiveness of SRS in treating cerebral AVMs and provides valuable insights into factors affecting treatment outcomes. The findings suggest that patients can expect a high chance of successful obliteration of the AVM with minimal adverse effects, making SRS a compelling option for those affected by this condition.

## Introduction

Cerebral arteriovenous malformations (AVM) are uncommon vascular anomalies in the brain, typically present from birth, with about 0.05% of people having an AVM [1]. The yearly risk of hemorrhage due to AVMs is roughly estimated to fall between 2 and 4%. AVMs within the brain can lead to several long-term complications such as seizures, neurological impairments, and brain bleeding. The likelihood of these events is determined by the Brown formula, which calculates risk as a percentage using the equation 105 minus the patient’s age [2].

Treatment options for AVM include monitoring, surgical excision, radiosurgery, endovascular embolization, or a combination of therapies, depending on the risk of bleeding, which is influenced by various factors like the AVM’s size, location, and specific characteristics of its blood vessels. The risk of bleeding varies widely, from 0.9–34.3% per year, and is higher in cases of small size, deep location, high blood flow, and specific venous drainage patterns [3–6].

Most of the existing literature concerning AVMs treated via SRS features studies employing the Gamma Knife device (Elekta, AB, Stockholm, Sweden). Despite the widespread availability of this technology, there exists a paucity of data regarding the utilization of linear accelerator (LINAC) SRS for AVM treatment. However, advancements in LINAC-based SRS techniques over time have led to its increased adoption. As per Niccolato, obliteration rates ranging from 80–90% are observed in carefully selected cases with AVM lesions smaller than 3 cm [5]. Conversely, findings from the Zabel-du Bois group suggest that outcomes following stereotactic irradiation of larger AVMs are less favorable, typically achieving success rates between 43 and 70% [6]. The available literature underscores the necessity for further investigation to establish clear indications for embolization preceding radiosurgery, given conflicting reports regarding its efficacy and potential adverse effects.

Therefore, there is a pressing need for additional data to elucidate the efficacy and therapeutic boundaries of SRS for cerebral AVMs. This study endeavors to scrutinize our institution’s outcomes concerning LINAC-based SRS for cerebral AVMs and contextualize them within the broader body of relevant literature. The objective of this study was to evaluate the outcomes of cerebral AVM treatment using LINAC-based SRS and to compare our findings to the existing literature on the subject.

## Methods and materials

### Patients

We performed a retrospective analysis for our medical archive, and we identified patients who underwent SRS for AVM and had a minimum follow-up period of 3 months. All patients were discussed in our multidisciplinary team (MDT) comprising neurosurgeons, neuroradiologists, and radiation oncologists. Patients considered nonviable candidates for microsurgery or embolization were subsequently addressed in the SRS MDT. All patients were treated at our institution in Marburg. Comprehensive post-SRS imaging, encompassing digital subtraction angiography (DSA) and magnetic resonance imaging (MRI), as soon as routinely available, was systematically reviewed. Collaboration between a neuroradiologist and a radiation oncologist facilitated this assessment. On DSA, complete obliteration was defined as the total disappearance of the AVM nidus and the associated feeding channels, establishing it as the gold standard.

### Irradiation technique

Radiotherapy was planned utilizing computed tomography (CT) imaging with contrast, mostly in conjunction with magnetic resonance imaging (MRI; 1–2 mm slice thickness), and guided by stereotactic methods employing a minimally invasive stereotactic frame. The stereotactic frame was affixed to the CT and radiotherapy couch to ascertain the isocenter across the modalities. Additionally, standard biplane DSA was utilized to aid in contouring the target volume for radiotherapy, encompassing the entire arteriovenous malformation nidus. Radiotherapy planning utilized a three-dimensional planning system, with target volume delineation performed via manual segmentation [7]. Radiotherapy was delivered using a linear accelerator emitting 6 MV X-rays. The position of the isocenter was verified prior to the start of irradiation. The dose was prescribed to the 80% isodose line, encompassing the entire nidus as well as previously embolized regions. All patients were treated with a prescribed dose of 20 Gy to the 80% isodose line using single fraction SRS, except for those who underwent hypofractionated treatment. Due to the unavailability of exact AVM volume measurements, the target volume was defined anatomically as the AVM nidus as delineated on MRI and angiographic imaging. A planning target volume (PTV) margin of 0–2 mm was applied, depending on image quality and proximity to critical structures.

Beam shaping was achieved using a micro-multileaf collimator with a leaf width of 2 mm at the isocenter. To minimize the risk of postradiosurgical cerebral edema, patients received a single intravenous dose of 20 mg dexamethasone prior to irradiation. Discharge typically occurred on the first postoperative day following SRS.

All treatment decisions were made following presentation of cases at the weekly in-house interdisciplinary neuro-oncology board. Stereotactic radiosurgery was considered in cases where patients presented with deep brain and/or unresectable AVMs, AVMs located in high-risk functional areas, or cases where previous embolization had failed to achieve complete AVM occlusion using MRI (determined as significant obliteration [SO]). Significant obliteration was defined as a visibly marked reduction of the nidus on follow-up MRI or angiography, without evidence of active shunting, but not yet meeting the criteria for complete obliteration. Patients exhibiting complete obliteration (CO) based on MRI-angiography underwent subsequent DSA for confirmation. Patients demonstrating CO on DSA were followed up every 2 years.

### Follow-up

The initial clinical assessment occurred 4–6 weeks posttreatment, with the first MRI control performed 3 months after irradiation. Subsequently, follow-up MRI was conducted every 6 months.

Annual control examinations were instituted upon confirmation of obliteration. Imaging assessments following treatment were discontinued upon obliteration diagnosis. The evaluation of obliteration was conducted through either MRI or DSA.

### Statistical analysis

Actuarial obliteration rates were assessed using Kaplan–Meier survival analysis with log-rank tests for group comparisons. Annual cumulative bleeding rates were calculated using Kaplan–Meier life tables. Univariate Cox proportional hazards regression analyzed factors affecting obliteration rates. All calculations were performed using SPSS 29.0 (IBM Corp., Armonk, NY, USA), with significance set at *p* < 0.05.

## Results

### Patient characteristics

In our study, a total of 134 patients who underwent radiosurgery between January 1983 and October 2022 were identified. Among these, 134 patients presented with a solitary AVM. The 3 patients who received hypofractionated stereotactic radiotherapy (HSRT) constituted a very small proportion of the overall cohort and were not expected to exert a statistically relevant influence on the outcome measures. Their inclusion or exclusion does not materially affect the overall conclusions of the study. The median age at the time of radiosurgery was 35 years (range 5–73 years, interquartile range [IQR] 24–48 years), with 21 patients under 18 years of age (pediatric patients defined as < 18 years). The male-to-female ratio was 1.09:1, comprising 70 males and 64 females (Table 1).

**Table 1:** Patient characteristics

Variable		Range
Gender	Male 70 Female 64	–
Age, years	Median 35	7–78
Maximum diameter, mm	1.82	0.4–6.7
Volume of lesion, cc	Median 4.64	0.04–22.7
Time to obliterate	Median 40	7–162
Pre-SRS DSA	79	–
Pre-SRS operation	7	–
Pre-SRS hemorrhage	60	–
Pre-SRS seizures	38	–
Pre-SRS headache	44	–
Pre-SRS visual deficit	22	–
Pre-SRS neurologic deficit	43	–
Post-SRS bleeding	8	–
Post-SRS seizures	8	–
Post-SRS vertigo	13	–
Post-SRS headache	29	–
Post-SRS hair loss	4	–

*SRS* stereotactic radiosurgery, *DSA* digital subtraction angiography

**Fig. 1:**
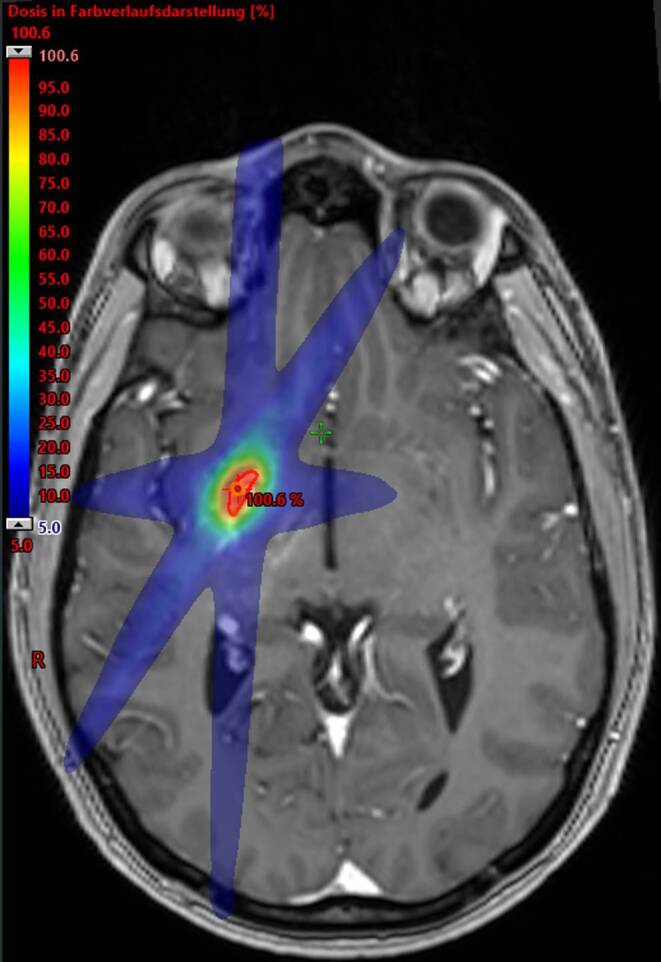
Radiosurgery plan with 20 Gy (80% isodose)

Preradiosurgery symptoms varied among patients: 44.8% (60/134) experienced hemorrhage, 28.4% (38/134) exhibited epilepsy, 16.4% (22/134) had visual deficits, 32.1% (43/134) presented with focal neurologic deficits, and 32.8% (44/134) reported headaches. The mean AVM volume was 4.64 ccm (range 0.04–22 ccm, IQR 2.1–6.8 ccm). The median radiosurgery dose was 20 Gy (range 14–25; IQR 18–22 Gy) (Fig. 1). The radiosurgery was predominantly administered in a single fraction, with only 3 patients receiving a fractionated SRS. Dose prescription was to the 80% isodose line in 131 patients (97.8%) with a median dose of 20 Gy, while 3 patients (2.2%) were treated to the 90% isodose line due to proximity to critical structures. Treatment regimens included 3 fractions of 7 Gy (total dose: 21 Gy), 5 fractions of 5 Gy (25 Gy total), and 5 fractions of 6 Gy (30 Gy total), respectively.

Spetzler–Martin (SM) grade distribution: grade I (7 patients, 5.2%), grade II (46 patients, 34.3%), grade III (51 patients, 38.1%), grade IV (22 patients, 16.4%), and grade V (8 patients, 6.0%). Mean SM grade was 2.76.

Prior interventions included the following: partial embolization (50 patients, 37.3%), combined embolization and surgical resection (8 patients, 6.0%), surgery alone (1 patient, 0.7%), and no prior treatment (75 patients, 56.0%).

## Outcomes

### Obliteration

**Fig. 2:**
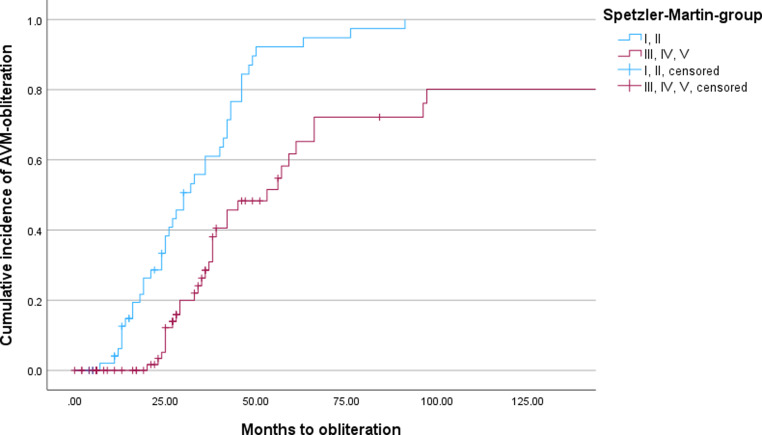
Kaplan–Meier curve analyses illustrating time to arteriovenous malformation (AVM) obliteration after stereotactic radiosurgery (SRS) and differences between groups 1 (Spetzler–Martin [SM] I and II) and 2 (SM III, IV, and V)

**Fig. 3:**
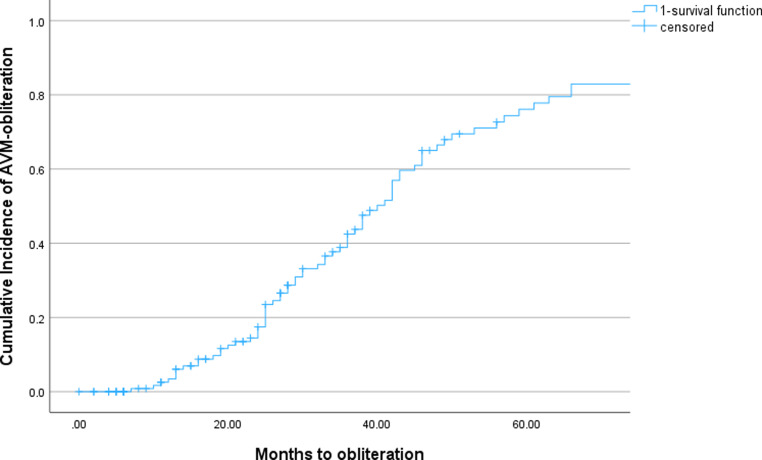
Kaplan–Meier curve analyses illustrating time to arteriovenous malformation (AVM) obliteration after stereotactic radiosurgery (SRS) for the whole cohort

Actuarial obliteration rates at 1, 2, 3, 4, and 5 years were 3.4%, 17.5%, 42.5%, 66.5%, and 76.9%, respectively. With mean follow-up of 75.9 months (range 2–371 months, standard deviation [SD] 72.4, median 48 months), 75 patients (56%) achieved complete obliteration, while 39 patients (29.0%) showed lesion regression. In all, 20 patients (15.3%) demonstrated no radiological progress after SRS (Figs. 2 and 3).

Median time to radiological obliteration was 36 months (range 7–162 months, IQR 24–54 months). Higher SM grade demonstrated significant association with reduced obliteration in univariate Cox regression analysis (hazard ratio [HR] 1.45, 95% confidence interval [CI] 1.08–1.94, *p* = 0.01).

SM grade-specific 5‑year obliteration rates were the following: grade I–II: 85.2%, grade III: 76.4%, and grade IV–V: 62.1%. Age, sex, and pediatric status did not significantly influence AVM response to SRS. Prior embolization showed no significant impact on obliteration rates (*p* = 0.34) (Tables 2 and 3).

**Table 2:** Association of Spetzler Martin grades and obliteration rate

Spetzler–Martin	Median time	Standard deviation	Confidence interval
1	25	6.6	11.9–38
2	32	3.9	24.4–39.5
3	39	2.4	34.3–42.7
4	97	25.9	46.1–147.9

**Table 3:** Factors affecting the obliteration

Factor	*p* value	95% CI
Age	0.845	−0.008 to 0.19
Child	0.997	−0.008 to 0.008
Sex	0.031	−0.008 to 0.106
SM	< 0.001	0.036–0.237

*SM* Spetzler Martin grade, *95% CI* 95% confidence interval

### Complications of treatment

No treatment-related mortality occurred. Late therapy-associated adverse events (> 6 months) were rare. No radiation necrosis was observed in our cohort. New permanent neurological deficits such as hemiparesis, sensory impairments, or memory dysfunction were not recorded.

Temporary side effects included headaches (28/134, 20.9%), hair loss (4/134, 3.0%), dizziness (13/134, 9.7%), and seizures (8/134, 6.0%). Post-SRS seizures were effectively managed with antiepileptic drugs. Transient scalp numbness from stereotactic pins and temporary radiation-induced alopecia were observed but resolved completely.

Imaging abnormalities such as perilesional edema were identified in 44 patients (32.8%), consistent with previous literature reporting rates up to 50%.

In all, 9 patients (6.9%) suffered intracranial hemorrhage after SRS during follow-up (range 5–188 months posttreatment). Annual cumulative bleeding rates were 1.5% for the first year and 0.7% for the second year. Among bleeding patients, 5 had previous hemorrhage, 5 underwent prior partial embolization, and 3 had additional microsurgical procedures before SRS. Mean irradiation dose for bleeding patients was 18.9 Gy. Two patients required only conservative management, while 7 required interventions including external ventricular drainage, ventriculoperitoneal shunt insertion, or microsurgical procedures.

## Discussion

This comprehensive analysis underscores the significant therapeutic potential and effectiveness of LINAC-based stereotactic radiosurgery across a broad spectrum of patients. The procedure demonstrates a low incidence of toxicity, regardless of demographic variables like age, sex, lesion location, or pediatric cases. The rates of arteriovenous malformation obliteration following SRS vary widely across studies, primarily influenced by treatment dose and AVM dimensions, with the Spetzler–Martin grading system emphasizing size as a critical determinant of outcomes.

A pivotal study by Flickinger et al. [8] explored the relationship between dose intensity and AVM treatment results, achieving a complete obliteration rate of 73% among 264 patients treated with Gamma Knife SRS. Their data suggested that obliteration rates peaked at 88% when doses reached 25 Gy. Similarly, Friedman et al. [9] analyzed 158 cerebral AVM cases managed using LINAC-SRS and reported obliteration rates of 81% for lesions between 1 and 4 cm^3^, 89% for those between 4 and 10 cm^3^, and 69% for AVMs exceeding 10 cm^3^. In our cohort of 134 patients treated with LINAC-SRS, actuarial obliteration rates reached 17.5% at 2 years and 42.5% at 3 years, with an average time to obliteration of 36 months (range 7–162 months). These findings are marginally below reported values in prior research, likely due to the larger average lesion size and higher mean Spetzler–Martin grades (2.76) in our cohort. Nonetheless, our data align closely with the findings of Zabel-du Bois et al. [10], who observed actuarial obliteration rates of 50% at 3 years in 65 patients, with an average obliteration time of 22.4 months.

The mean nidus volume in this study was 4.64 cm^3^, comparable to the 5.2 cm^3^ reported by Zabel-du Bois et al., but smaller than the 7.1 cm^3^ documented in other analyses. The mean dose delivered in our cohort was 20.0 Gy to the 80% isodose line, which could account for the favorable outcomes relative to studies applying lower peripheral doses. AVMs carry an annual hemorrhage risk of 2–4%, increasing to approximately 6% in the year following an initial bleed before declining thereafter. Sasaki et al. reported an annual rebleeding risk of 13%, with morbidity and mortality rates of 12.5 and 62.5%, respectively, underscoring the urgency of timely intervention to mitigate recurrent hemorrhage risks. The optimal treatment approach for large AVMs, particularly in patients with unruptured lesions, remains uncertain. Findings from the ARUBA trial (A Randomized Trial of Unruptured Brain Arteriovenous Malformations) indicated that conservative management may be preferable to interventional therapies [11]. However, patients managed conservatively may still face an increased risk of neurological complications, and some experience significant psychological distress, including tension and anxiety, which can impair their quality of life. Therefore, careful patient selection is essential to determine who may benefit from interventional treatment (Table 4).

**Table 4:** Recent studies of linac-based stereotactic radiosurgery (SRS) for arteriovenous malformations (AVM)

Study	*N*	Obliteration rate (%)	Dose (median)	Treatment volume, cc	Reported toxicity (%)	Follow-up (median)
Bollet et al. [12]	118	54	24.5 Gy	7.4	3.9	3.8
Zabel Du Bois et al. [13]	50	76	18 Gy	4	12	3.1
Gobin et al. [14]	125	65	25 Gy	6.2	3	3.3
Miyawaki et al. [4]	73	50	25.18 Gy/max	8.4	22	5.9
Yahya et al. [15]	47	74.5	19.8 Gy	1.97	6.4	4.4
Schlienger et al. [16]	169	64	25 Gy	2.46	2.3	4.8
Gawish et al. [17]	68	68	19.8 Gy	10.6	20	2.1

*N* number of patients included, *cc* cubic centimeters

Research by Zabel-du Bois et al. revealed a progressive decline in post-SRS hemorrhage risk, with rates of 4.7%, 3.4%, and 2.7% in the first 3 years, respectively. In our study, a hemorrhage rate of 6.7% was observed, further corroborating the trend of reduced bleeding risk after SRS. Nine patients in our cohort (6.7%) experienced intracranial hemorrhages within a follow-up range of 5 to 188 months posttreatment. Despite these events, no additional neurological deficits were detected, and most patients had undergone prior embolization or surgery before SRS, highlighting the benefits of an interdisciplinary approach.

Our findings indicate that the latency period for AVM obliteration can extend beyond the usual 2–3 years, particularly for larger lesions. This observation mirrors results from Touboul et al., who documented obliteration rates of 40% at 3 years and 62% at 5 years in a study of 100 patients. Similarly, Zabel-du Bois et al. noted an increase from 47% at 3 years to 60% at 4 years.

An evaluation of the impact of prior embolization on obliteration rates showed no substantial differences between embolized and nonembolized AVMs, contradicting earlier studies that suggested embolization might reduce the efficacy of SRS. Our approach involves targeting the entire nidus to ensure uniform dose delivery, potentially preventing revascularization and offsetting the proangiogenic effects of embolization [17, 18]. This finding supports the use of SRS as an effective salvage treatment following incomplete embolization or as part of a multimodal treatment strategy.

Blamek et al. [19] emphasized that doses under 15 Gy significantly reduce obliteration success. Nataf et al. [20] observed obliteration rates of 44% with a dose of 15 Gy and 89% with doses between 15 and 20 Gy. Our study reported minimal adverse effects, with 2 patients experiencing transient neurological symptoms that resolved within weeks. Importantly, no cases of radiation necrosis were observed in our patient cohort. Imaging abnormalities such as edema or necrosis were identified in 33.9% of cases, aligning with previous findings indicating rates as high as 50% [21–23].

Among the pediatric subset, comprising 15.7% of our cohort, angiographic obliteration was achieved in 52.4% of cases, with no observed hemorrhages. While the sample size limited subgroup analysis, larger studies like Kano et al. [24] demonstrated a 67% obliteration rate at 5 years in 135 pediatric patients, with an annual latency-period bleeding risk of 1.8%. AVMs located in the posterior fossa present a higher risk of latent hemorrhage, as noted by Shin et al., necessitating extended monitoring and individualized treatment plans for this subset [6, 25].

The group exhibited considerable heterogeneity, and the radiation doses were mild, averaging 19.0 Gy, which may be regarded as low to achieve optimal therapeutic efficacy. Reductions in dosage arise from substantial AVM volumes and provide the danger of harmful consequences linked to administering elevated doses to extensive brain volumes. Laakso et al. [26] indicate that even partial therapy decreases mortality in individuals with arteriovenous malformations (AVMs) and may be appropriate for surgical intervention. Neoadjuvant radiation therapy can enhance surgical procedures and diminish morbidity. Sanchez-Mejia et al. [27] suggested that follow-up examinations should be prolonged to enable a more accurate evaluation of treatment concerning bleeding risk, long-term obliteration rate, and AVM-related mortality.

## Conclusion

This study expands the knowledge base regarding the utility of stereotactic radiosurgery (SRS) as a therapeutic option for intracerebral arteriovenous malformation (AVMs). Overall, there is a low morbidity rate and a high likelihood of nidus obliteration. Treatment dose and the largest nidus diameter were the major factors influencing obliteration. Larger AVMs should be additionally considered, as they exhibit a lower obliteration rate regardless of whether they are treated with radiosurgery or fractionated radiotherapy.

## References

[1] Galarza M et al (2014) Jazz, guitar, and neurosurgery: the Pat Martino case report. World Neurosurg 81:651.E1–651.E7 24076057 10.1016/j.wneu.2013.09.042

[2] Brown RD (2000) Simple risk predictions for arteriovenous malformation hemorrhage [1] (Multiple Letters). Neurosurgery 10764285

[3] Hernesniemi JA, Dashti R, Juvela S, Väärt K, Niemelä M, Laakso A (2008) Natural history of brain arteriovenous malformations: a long-term follow-up study of risk of Hemorrhage in 238 patients. Neurosurgery 10.1227/01.NEU.0000330401.82582.5E 19005371

[4] Miyawaki L, Dowd C, Wara W, Goldsmith B, Albright N, Gutin P, Halbach V, Hieshima G, Higashida R, Lulu B, Pitts L, Schell M, Smith V, Weaver K, Wilson C, Larson D (1999) Five year results of LINAC radiosurgery for arteriovenous malformations: outcome for large AVMS. Int J Radiat Oncol Biol Phys 10.1016/s0360-3016(99)00102-9 10421543

[5] Nicolato A, Lupidi F, Sandri MF, Foroni R, Zampieri P, Mazza C, Maluta S, Beltramello A, Gerosa M Gamma knife radiosurgery for cerebral arteriovenous malformations in children/adolescents and adults. Part 10.1016/j.ijrobp.2005.07.983 16257134

[6] II (2006) differences in obliteration rates, treatment-obliteration intervals, and prognostic factors. Int J Radiat Oncol Biol Phys 10.1016/j.ijrobp.2005.09.013 16338096

[7] Mast H, Young WL, Koennecke H‑C et al (1997) Risk of spontaneous hemorrhage after diagnosis of cerebral arteriovenous malformation. Lancet 350:1065–1068 10213548 10.1016/s0140-6736(97)05390-7

[8] Karlsson B, Lindquist C, Steiner L (1997) Prediction of obliteration after gamma knife surgery for cerebral arteriovenous malformations. Neurosurgery 40(1997):425–431 9055280 10.1097/00006123-199703000-00001

[9] Flickinger JC, Kondziolka D, Maitz AH et al (2002) An analysis of the dose-response for arteriovenous malformation radiosurgery and other factors affecting obliteration. Radiother Oncol 63:347–354 12142099 10.1016/s0167-8140(02)00103-2

[10] Friedman WA, Bova FJ, Mendenhall WM (1995) Linear accelerator radiosurgery for arteriovenous malformations: the relationship of size to outcome. J Neurosurg 82:180–189 7815144 10.3171/jns.1995.82.2.0180

[11] Akakin A, Ozkan A, Akgun E, Koc DY, Konya D, Pamir MN et al (2010) Endovascular treatment increases but gamma knife radiosurgery decreases angiogenic activity of arteriovenous malformations: an in vivo experimental study using a rat cornea model. Neurosurgery 66(1):121–129 (discussion 129–130) 20023542 10.1227/01.NEU.0000363154.88768.34

[12] Sanchez-Mejia RO, McDermott MW, Tan J, Kim H, Young WL, Lawton MT (2009) Radiosurgery facilitates resection of brain arteriovenous malformations and reduces surgical morbidity. Neurosurgery 64(2):231–238 19057424 10.1227/01.NEU.0000338068.44060.EA PMC2893586

[13] Pollock BE, Flickinger JC (2008) Modification of the radiosurgery based arterio-venous malformation grading system. Neurosurgery 63(2):239–243 18797353 10.1227/01.NEU.0000315861.24920.92

[14] Schlienger M, Atlan D, Lefkopoulos D et al (2000) Linac radiosurgery for cerebral arteriovenous malformations: results in 169 patients. Int J Radiat Oncol Biol Phys 46(5):1135–1142 10725623 10.1016/s0360-3016(99)00523-4

[15] Bollet MA, Anxionnat R, Buchheit I et al (2004) Efficacy and morbidity of arc-therapy radiosurgery for cerebral arteriovenous malformations: a comparison with the natural history. Int J Radiat Oncol Biol Phys 58(5):1353–1363 15050310 10.1016/j.ijrobp.2003.09.005

[16] Yamamoto M, Jimbo M, Kobayashi M et al (1992) Long-term results of radio-surgery for arteriovenous malformation: neuroradiodiagnostic imaging and histological studies of angiographically confirmed nidus obliteration. Surg Neurol 37:219–230 1536028 10.1016/0090-3019(92)90235-f

[17] Yahya S, Heyes G, Nightingale P, Lamin S, Chavda S, Geh I, Spooner D, Cruickshank G, Sanghera P (2017) Linear accelerator radiosurgery for arteriovenous malformations: updated literature review. J Clin Neurosci 38:91–95. 10.1016/j.jocn.2016.12.015 28117260 10.1016/j.jocn.2016.12.015

[18] Gawish A, Röllich B, Ochel HJ, Brunner TB (2022) Linac-based stereotactic radiosurgery for brain arteriovenous malformations. Radiat Oncol 17(1):161 36175931 10.1186/s13014-022-02130-2 PMC9520913

[19] Pollock BE, Gorman DA, Brown PD (2004) Radiosurgery for arteriovenousn malformations of the basal ganglia, thalamus and brainstem. J Neurosurg 100:210–214 15086226 10.3171/jns.2004.100.2.0210

[20] Blamek S, Tarnawski R, Miszczyk L (2011) Linac-based stereotactic radiosurgery for brain arteriovenous malformations. Clin Oncol 23(8):525–531. 10.1016/j.clon.2011.03.012 10.1016/j.clon.2011.03.012 21501954

[21] Nataf F, Schlienger M, Lefkopoulos D et al (2003) Radiosurgery of cerebral arte-riovenous malformations children: a series of 57 cases. Int J Radiat Oncol Biol Phys 57(1):184–195 12909232 10.1016/s0360-3016(03)00445-0

[22] Blamek S, Boba M, Larysz D et al (2010) The incidence of imaging abnormalities after stereotactic radiosurgery for cerebral arteriovenous and cavernous malformations. Acta Neurochir Suppl 106:187–190 19812946 10.1007/978-3-211-98811-4_34

[23] van den Berg R, Buis DR, Lagerwaard FJ, Lycklama A, Nijeholt GJ, Vander-top WP (2008) Extensive white matter changes after stereotactic radiosurgery for brain arteriovenous malformations: a prognostic sign for obliteration? Neurosurgery 63(6:1064–1069 19008768 10.1227/01.NEU.0000330413.73983.02

[24] Flickinger JC, Kondziolka D, Lunsford LD et al (1999) A multi-institutional analysis of complication outcomes after arteriovenous malformation radiosurgery. Int J Radiat Oncol Biol Phys 44(1):67–74 10219796 10.1016/s0360-3016(98)00518-5

[25] Kano H, Kondziolka D, Flickinger J et al (2012) Stereotactic radiosurgery for arteriovenous malformations, part 2: management of pediatric patients. J Neurosurg Pediatr 9:1–10 22208313 10.3171/2011.9.PEDS10458

[26] Shin M, Kawamoto S, Kurita H et al (2002) Retrospective analysis of a 10-year experience of stereotactic radiosurgery for arteriovenous malformations in children and adolescents. J Neurosurg 97:779–784 12405363 10.3171/jns.2002.97.4.0779

[27] Laakso A, Dashti R, Seppänen J et al (2008) Long-term excessmortality in 623 patients with brain arteriovenous malformations. Neurosurgery 63(2):244–253 18797354 10.1227/01.NEU.0000320439.27895.24

[28] Chang TC, Shirato H, Aoyama H, Ushikoshi S, Kato N, Kuroda S, Ishikawa T, Houkin K, Iwasaki Y, Miyasaka K (2004) Stereotactic irradiation for intracranial arteriovenous malformation using stereotactic radiosurgery or hypof-ractionated stereotactic radiotherapy. Int J Radiat Oncol Biol Phys 10.1016/j.ijrobp.2004.04.041 15465204

[29] Zabel-du Bois A, Milker-Zabel S, Huber P, Schlegel W, Debus J (2006) Pediatric cerebral arteriovenous malformations: the role of stereotactic Linac-based radiosurgery. Int J Radiat Oncol Biol Phys 10.1016/j.ijrobp.2006.01.041 16682140

[30] Pollock BE, Flickinger JC (2002) A proposed radiosurgery-based grading system for arteriovenous malformations. J Neurosurg 96:79–85 11794608 10.3171/jns.2002.96.1.0079

[31] Zabel A, Milker-Zabel S, Huber P et al (2005) Treatment outcome after linac-based radiosurgery in cerebral arteriovenous malformations: Retrospective analysis of factors affecting obliteration. Radiother Oncol 77:105–110 15893833 10.1016/j.radonc.2005.04.008

[32] Zabel-du Bois A, Milker-Zabel S, Huber P, Schlegel W, Debus J (2006) Stereotactic linac-based radiosurgery in the treatment of cerebral arteriovenous malformations located deep, involving corpus callosum, motor cortex or brainstem. Int J Radiat Oncol Biol Phys 64(4):1044–1048 16373080 10.1016/j.ijrobp.2005.09.024

[33] Zabel-du Bois A, Milker-Zabel S, Huber P, Schlegel W, Debus J (2006) Linac-based radiosurgery or hypofractionated stereotactic radiotherapy in the treatment of large cerebral arteriovenous malformations. Int J Radiat Oncol Biol Phys 64(4):1049–1054 16376487 10.1016/j.ijrobp.2005.09.021

[34] Ondra SL, Troupp H, George ED et al (1990) The natural history of symptomatic arteriovenous malformations of the brain: a 24-year follow-up assessment. J Neurosurg 73:387–391 2384776 10.3171/jns.1990.73.3.0387

[35] Sasaki T, Kurita H, Saito I et al (1998) Arteriovenous malformations in the basal ganglia and thalamus: management and results in 101 cases. J Neurosurg 88:282–292 10.3171/jns.1998.88.2.0285 9452237

[36] Andrade-Souza YM, Zadeh G, Scora D et al (2005) Radiosurgery for basal ganglia, internal capsule, and thalamus arteriovenous malformation: clinical outcome. Neurosurgery 56:56–64 15617586 10.1227/01.neu.0000145797.35968.ed

[37] Zabel-du Bois A, Milker-Zabel S, Huber P, Schlegel W, Debus J (2007) Risk of hemorrhage and obliteration rates of LINAC-based radiosurgery for cerebral arteriovenous malformations treated after prior partial embolization. Int J Radiat Oncol Biol Phys 68(4):999–1003 17398029 10.1016/j.ijrobp.2007.01.027

[38] Touboul E, Al Halabi A, Buffat L et al (1998) Single-fraction stereotactic radiotherapy: a dose-response analysis of arteriovenous malformation obliteration. Int J Radiat Oncol Biol Phys 41(4):855–861 9652849 10.1016/s0360-3016(98)00115-1

[39] Gobin YP, Laurent A, Merienne L et al (1996) Treatment of brain arterio-venous malformations by embolization and radiosurgery. J Neurosurg 85(1):19–28 8683274 10.3171/jns.1996.85.1.0019

[40] Andrade-Souza YM, Ramani M, Scora D, Tsao terBrugge MN, terBrugge K, Schwartz ML (2007) Embolization before radiosurgery reduces the obliteration rate of arteriovenous malformations. Neurosurgery 60(3):443–451 17327788 10.1227/01.NEU.0000255347.25959.D0

[41] Mohr JP, Overbey JR, Hartmann A, Kummer RV, Al-Shahi SR, Kim H, van der Worp HB, Parides MK, Stefani MA, Houdart E, Libman R, Pile-Spellman J, Harkness K, Cordonnier C, Moquete E, Biondi A, Klijn CJM, Stapf C, Moskowitz AJ (2020) ARUBA co-investigators. Medical management with interventional therapy versus medical management alone for unruptured brain arteriovenous malformations (ARUBA): final follow-up of a multicentre, non-blinded, randomised controlled trial. Lancet Neurol 19(7):573–581. 10.1016/S1474-4422(20)30181-2 (PMID: 32562682.) 32562682 10.1016/S1474-4422(20)30181-2

